# Topological Analysis of MAPK Cascade for Kinetic ErbB Signaling

**DOI:** 10.1371/journal.pone.0001782

**Published:** 2008-03-12

**Authors:** Takashi Nakakuki, Noriko Yumoto, Takashi Naka, Mikako Shirouzu, Shigeyuki Yokoyama, Mariko Hatakeyama

**Affiliations:** 1 Cellular Systems Biology Team, Computational and Experimental Systems Biology Group, RIKEN Genomic Sciences Center, Tsurumi-ku, Yokohama, Kanagawa, Japan; 2 Department of Intelligent Informatics, Faculty of Information Science, Kyushu Sangyo University, Higashi-ku, Fukuoka, Japan; 3 Protein Research Group, RIKEN Genomic Sciences Center, Tsurumi-ku, Yokohama, Kanagawa, Japan; 4 Department of Biophysics and Biochemistry, Graduate School of Science, The University of Tokyo, Bunkyo-ku, Tokyo, Japan; IBM Thomas J. Watson Research Center, United States of America

## Abstract

Ligand-induced homo- and hetero-dimer formation of ErbB receptors results in different biological outcomes irrespective of recruitment and activation of similar effector proteins. Earlier experimental research indicated that cells expressing both EGFR (epidermal growth factor receptor) and the ErbB4 receptor (E1/4 cells) induced E1/4 cell-specific B-Raf activation and higher extracellular signal-regulated kinase (ERK) activation, followed by cellular transformation, than cells solely expressing EGFR (E1 cells) in Chinese hamster ovary (CHO) cells. Since our experimental data revealed the presence of positive feedback by ERK on upstream pathways, it was estimated that the cross-talk/feedback pathway structure of the Raf-MEK-ERK cascade might affect ERK activation dynamics in our cell system. To uncover the regulatory mechanism concerning the ERK dynamics, we used topological models and performed parameter estimation for all candidate structures that possessed ERK-mediated positive feedback regulation of Raf. The structure that reliably reproduced a series of experimental data regarding signal amplitude and duration of the signaling molecules was selected as a solution. We found that the pathway structure is characterized by ERK-mediated positive feedback regulation of B-Raf and B-Raf-mediated negative regulation of Raf-1. Steady-state analysis of the estimated structure indicated that the amplitude of Ras activity might critically affect ERK activity through ERK-B-Raf positive feedback coordination with sustained B-Raf activation in E1/4 cells. However, Rap1 that positively regulates B-Raf activity might be less effective concerning ERK and B-Raf activity. Furthermore, we investigated how such Ras activity in E1/4 cells can be regulated by EGFR/ErbB4 heterodimer-mediated signaling. From a sensitivity analysis of the detailed upstream model for Ras activation, we concluded that Ras activation dynamics is dominated by heterodimer-mediated signaling coordination with a large initial speed of dimerization when the concentration of the ErbB4 receptor is considerably high. Such characteristics of the signaling cause the preferential binding of the Grb2-SOS complex to heterodimer-mediated signaling molecules.

## Introduction

Overexpression or mutation of the ErbB receptor is closely correlated with the incidence of various kinds of human cancer [1], [2]. The risk of cancer becomes especially elevated when different ErbB receptors are co-expressed [3], [4]. This phenomenon is also confirmed at the cellular level, where transformation of cells occurs when different ErbB receptors are co-expressed in the same cells [5]–[7]. However, this cellular transformation mechanism has not been identified because an investigation of the primary interaction of adaptor proteins following kinase activation induced by growth hormones results in relatively small differences in protein binding patterns for cells expressing either single- or multiple-species of ErbB receptors [8]–[10]. For example, EGF (epidermal growth factor)-stimulated EGFR (epidermal growth factor receptor) in ErbB4 co-expressing cells essentially interacts with adaptor and effector proteins such as growth factor receptor-bound protein2 (Grb2), Src homology and collagen domain protein (Shc), the p85 subunit of phosphatidylinositol 3′-kinase (PI3K), Cbl and phospholipase Cγ (PLCγ) in a manner that is similar to EGF-stimulated cells solely expressing EGFR [8], [10]. Therefore, the increase in biological response elicited by the coexpression of ErbB receptors cannot be solely explained by specific protein interactions induced by each receptor. Recently, it was understood that quantitative (strength and duration of activities of the pathways) rather than qualitative (e.g. regulation of different pathways) differences between signaling pathways may largely account for dissimilar biological responses [11]. This explanation may be relevant to a general investigation of factors determining ligand-specific or receptor-specific signal transduction pathways when considering the fact that mammalian cells share almost the same sets of signaling components [12]–[14].

Several studies indicated that signal amplitude and duration are temporally modulated by cross-talk between two pathways (e.g. Raf inhibition by Akt) [14], [15] and inhibitory feedback from ERK to Grb2-SOS complex formation [16], [17] and Raf [18]–[20]. Furthermore, our previous study indicated that Chinese hamster ovary (CHO) cells expressing both EGFR and ErbB4 receptors (E1/4 cells) induced specific B-Raf and higher ERK activation than cells solely expressing the EGFR receptor (E1 cells) and induced cellular transformation [21]. It is therefore understood that regardless of the same primary recruitment of effector proteins to each ErbB receptor dimer, the cells induce different structures of downstream regulatory pathways and that such differences might cause a change in the kinase activity level in relation to the cell fate determination process.

The aim of the present study is to give a mechanistic insight into how E1/4 cells induce a higher amplitude of ERK activity than E1 cells and how B-Raf is involved in ERK activation in an E1/4 cell-specific manner. Our experimental data showed that the inhibiting kinase activity of MEK (MAP kinase kinase) on ERK lowered the MEK response with EGF, indicating that there might exist positive feedback by ERK somewhere within upstream pathways. Furthermore, there are increasing reports of cross-talk between Raf-1 and B-Raf pathways [18]–[20], [22], [23]. However, the pathway structure of feedback/cross-talk in the MAPK cascade is not clear in our CHO cell lines. Therefore, we estimated the structure based on model parameter estimation examining which structures could reliably reproduce the experimental data with respect to the signal amplitude and duration of the signaling molecules. Although a main structure of MAPK cascade was originally analyzed by Heinrich et al. [24] and Huang et al. [25], we are the first to apply the topological modeling to CHO cells by adding cross-talk and feedback to explain ERK activation dynamics. As a result, we specified a structure that possesses both negative cross-talk regulation by B-Raf to Raf-1 and positive feedback by ERK to B-Raf. The steady-state analysis of the estimated pathway structure of the Raf-MEK-ERK cascade indicated that the amplitude of Ras activity might critically affect ERK activity through ERK-B-Raf positive feedback coordination with sustained B-Raf activation in E1/4 cells. On the other hand, it was shown that the amplitude of Rap1 activity might be less influential on ERK activity. Accordingly, we further investigated differences in amplitude of Ras activity in ErbB signaling pathways using a detailed upstream model modified from Kholodenko's [26]. The sensitivity analysis indicated that the initial reaction velocity of EGFR/ErbB4 receptor heterodimerization might be considerably higher than that of EGFR homodimerization, and as a result, the Grb2-SOS complex preferentially binds to the activated heterodimer rather than the activated homodimer. Finally, we concluded that the amplitude of Ras activity becomes more potent under high expression of the ErbB4 receptor in E1/4 cells.

## Results

### Estimation of the feedback and cross-talk structure of the Raf-MEK-ERK cascade in CHO cells using a mathematical model

**Figure 1:**
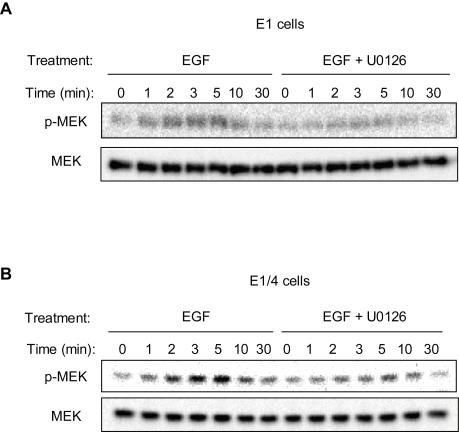
The effect of MEK inhibitor U0126 on MEK phosphorylation in E1 and E1/4 cells. Serum-starved E1 and E1/4 cells were incubated with 10 nM EGF for the indicated time period with or without pretreatment of 200 nM U0126. MEK phosphorylation was analyzed by Western blot with the corresponding anti-phospho-specific MEK antibodies (upper panel), and then reblotted with an anti-MEK antibody (lower panel). (A) Western blot for E1 cells. (B) Western blot for E1/4 cells. Data show a representative figure of three independent experiments. Quantified data are available in Table S1 (no. 3).

**Figure 2:**
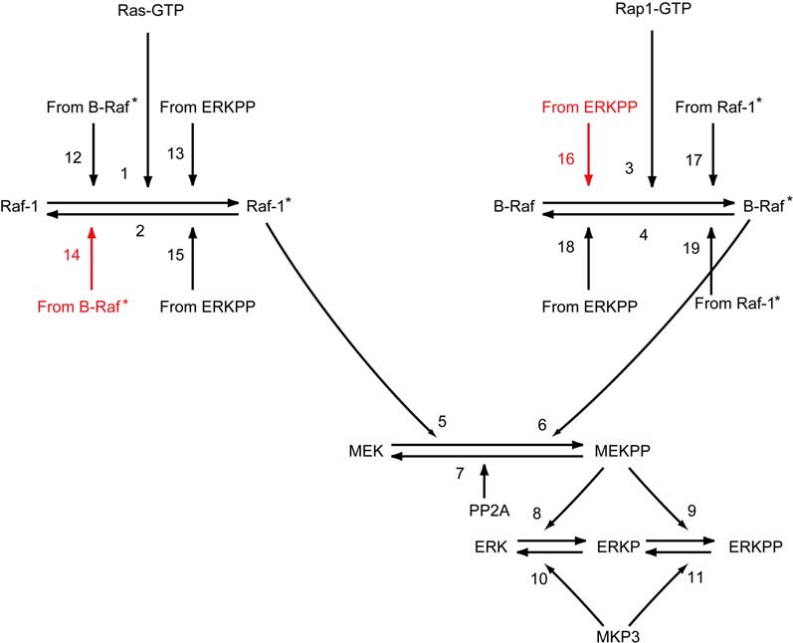
Topological model of the Raf-MEK-ERK cascade in CHO cells. The inputs of the Raf-MEK-ERK cascade are the activated forms of Ras and Rap1, Ras-GTP and Rap1-GTP. The output is the doubly phosphorylated ERK, ERKPP. Steps 1–11 are the fixed part of the cascade, whereas steps 12–19 are possible cross-talk/feedback connections to be determined. The selected pathways were steps 14 and 16 (red arrows). Numbers shown correspond to the kinetic equations in Table S5.

We first experimentally investigated the presence of feedback regulation by ERK on upstream signal transduction pathways using the MEK inhibitor. The MEK-mediated activation of ERK through phosphorylation of ERK threonine/tyrosine residues has been well established. The inhibition of kinase activity of MEK on ERK and subsequent measurement of MEK phosphorylation represents one approach that could be used to evaluate the effect of ERK-mediated feedback on the upstream pathways. U0126 is a selective inhibitor of MEK and can therefore be used to block the phosphorylation of ERK by MEK [27]. Results indicated that EGF-induced MEK activity decreased when ERK activation was inhibited through the use of U0126 in both E1 and E1/4 cells (Figure 1) (The Western blot data relating to ERK activity following U0126 use is provided in Figure S1). This fact implied positive feedback regulation by ERK on upstream molecules, although the feedback point was not clear. Additionally, there is a possibility of cross-talk between Raf isoforms, which has been reported in many cell lines [20], [22], [23]. In order to determine the structure of feedback/cross-talk in CHO cells, we carried out topological modeling of the central Raf-MEK-ERK cascade (Figure 2). We began by using the main structure of the MAPK cascade (steps 1–11) originally given by Heinrich et al. [24], and then included feedback (steps 13, 15, 16, 18), cross-talk (steps 12, 14, 17, 19), and dual phosphorylation of ERK (steps 8–11) components [28]–[30]. Detailed information pertaining to model construction is summarized in Materials and Methods. We assumed that the structure and kinetic parameters are identical for E1 and E1/4 cells, whereas time courses relating to Ras- and Rap1-GTPs, which represent inputs of the cascade, can differ. Moreover, we assumed that the effect of feedback regulation by ERK on upstream pathways can be approximated by the effect on Raf activity. For instance, suppose that SOS (Son of Sevenless homologue protein) is negatively regulated by ERK [16], and the down-regulation of SOS activity reduces Raf-1 activity via the down-regulation of Ras. Raf-1 activation is then modeled with ERK negative feedback in our topological model. Similarly, we assumed cross-talk regulation between Raf-1 and B-Raf.

**Figure 3:**
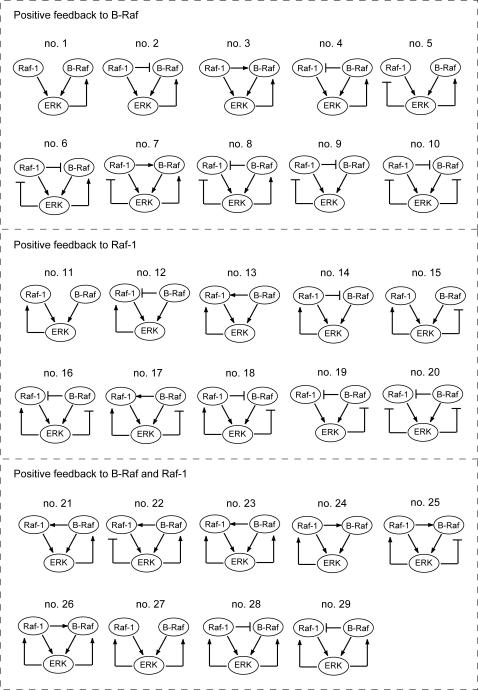
The 29 pathway structures that possess positive feedback by ERK to Raf. The 29 pathway structures that possess positive feedback by ERK to Raf isoforms can be classified into three types of regulation depending on which isoform is positively regulated: B-Raf (nos. 1–10), Raf-1 (nos. 11–20), or both B-Raf and Raf-1 (nos. 21–29). The symbol for MEK is omitted here for clarity.

Steps 12–19 were included as possible candidates of feedback/cross-talk connections that should be determined. The number of possible structures regarding cross-talk and feedback is 81 ( = 3^4^) since there can be three kinds of regulation (positive, negative, or not present) for each of four interactions, ERK to Raf-1 (steps 13 and 15), ERK to B-Raf (steps 16 and 18), Raf-1 to B-Raf (steps 17 and 19), and B-Raf to Raf-1 (steps 12 and 14). In order to narrow the candidates, we took into account the experimentally observed positive feedback by ERK (Figure 1). Moreover, for simplicity we excluded the situation involving the coexistence of both Raf-1 regulation by B-Raf and B-Raf regulation by Raf-1. These constraints narrowed down the 81 possible candidates to 29 structures that contain direct/indirect positive feedback by ERK to B-Raf (nos. 1–10), Raf-1 (nos. 11–20), and both B-Raf and Raf-1 (nos. 21–29) (Figure 3).

**Figure 4:**
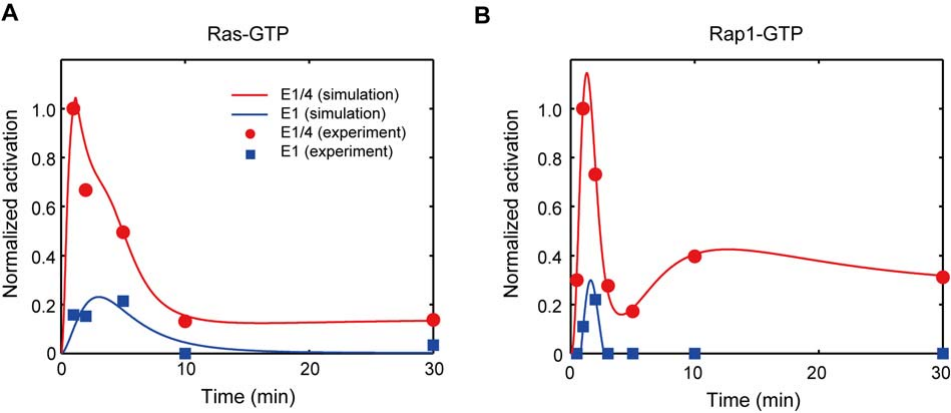
Time courses of Ras- and Rap1-GTPs. The input signal generator reproduced the time-course data of Ras- and Rap1-GTPs with 10 nM EGF. (A) Normalized time-course data of Ras-GTP. (B) Normalized time-course data of Rap1-GTP. The graphs represent the normalized activity of Ras- and Rap-1 GTPs in which the data are divided by the value for E1/4 cells at 1 min. Blue and red lines correspond to simulation data for E1 and E1/4 cells, respectively. The filled squares and circles correspond to experimental data concerning E1 and E1/4 cells, respectively.

For each of the 29 structures, we estimated the model parameters to reproduce the experimental data. Here, we employed the hypothesis that appropriate structures could explain experimental results using suitable kinetic parameters. Additionally, we also hypothesized that a biological pathway is robust against small parameter changes, and that the parameter should therefore be easily searched when the pathway structure is solid. For parameter estimation, we prepared 29 ODE (ordinary differential equation) models and six time-course datasets each from the Western blot analyses of E1 and E1/4 cells (Table 1, nos. 1–3). Additionally, we developed an input signal generator to reproduce the experimental data relating to Ras- and Rap1-GTPs (Figure 4 and Table 1, nos. 4 and 5). Detailed information relating to the input signal generator is provided in Text S1, Tables S2, S3, S4 and Figures S2 and S3. Moreover, we experimentally measured the time course of B-Raf activation (Table 1, no. 6), which showed sustained activation, for validation of the model. The procedure of model selection based on parameter estimation is provided in Materials and Methods.

**Table 1:** Time-course data from Western blot analysis.

No	Molecules	Inhibitor	data points (min)	treated cell lines	Intended purpose
1	phosphorylated ERK	-	0, 1, 2, 3, 5, 10, 20, 30	E1, E1/4	parameter estimation for the topological model
2	phosphorylated MEK	-	0, 1, 2, 3, 5, 10, 30	E1, E1/4	parameter estimation for the topological model
3	phosphorylated MEK	U0126	0, 1, 2, 3, 5, 10, 30	E1, E1/4	parameter estimation for the topological model
4	Ras-GTP	-	0, 1, 2, 5, 10, 30	E1, E1/4	training data for the input signal generator and the detailed upstream model
5	Rap1-GTP	-	0, 0.5, 1, 2, 3, 5, 10, 30	E1, E1/4	training data for the input signal generator and the detailed upstream model
6	activated B-Raf	-	0, 1, 2, 5, 10, 30	E1/4	additional test for the topological model
7	phosphorylated ERK	U73122	5	E1, E1/4	inhibitor test for the topological model
8	phosphorylated EGFR	-	0, 1, 2, 10, 30	E1, E1/4	parameter estimation for the detailed upstream model
9	phosphorylated ErbB4	-	0, 1, 2, 10, 30	E1/4	parameter estimation for the detailed upstream model
10	phosphorylated Shc	-	0, 1, 2, 5, 10, 30	E1, E1/4	parameter estimation for the detailed upstream model

The experimental protocol is summarized in Materials and Methods. The quantified data are shown in Table S1. All experiments were performed using 10 nM EGF.

**Figure 5:**
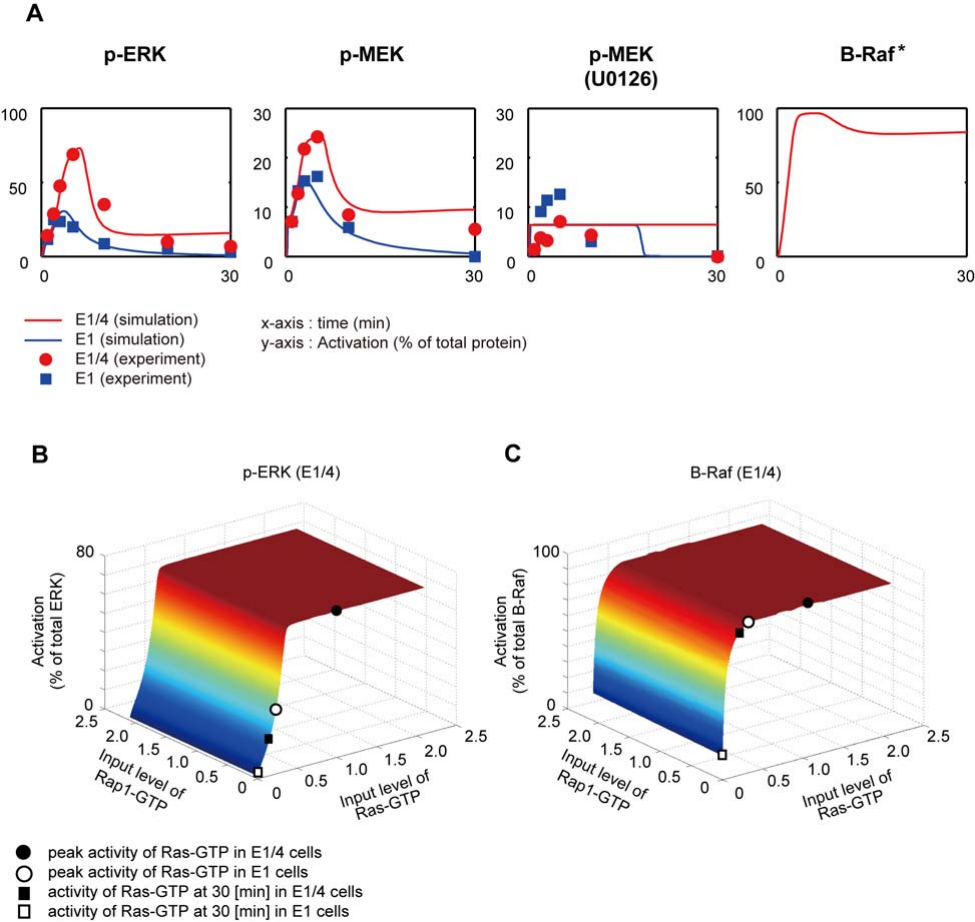
The time-course simulation and steady-state responses of the number 4 structure. (A) The simulation results are shown with the corresponding experimental data. Filled squares and circles indicate experimental data relating to E1 and E1/4 cells, respectively. Blue and red lines indicate the simulation data relating to E1 and E1/4 cells, respectively. The *x*-axis represents time (min) and the *y*-axis activation (% of total protein). In (B) and (C) the steady-state responses of ERK and B-Raf activity for the selected pathway structure are shown. The input signal levels of Ras- and Rap1-GTPs to the Raf-MEK-ERK cascade characterized by the number 4 structure were changed in the range 0.1 to 2.0. The simulation for each input level was then ran for 100,000 sec to approximate the steady-state values where the activity of all molecules hardly moved at about the termination time of the simulation. (B) 3-D graph of steady-state ERK activity. (C) 3-D graph of steady-state B-Raf activity. The colors of the graphs indicate activation (% of total protein). Open and filled circles correspond to the activation level induced by the input level equivalent to the peak level of Ras-GTP in E1 and E1/4 cells, respectively. Similarly, open and filled squares correspond to the activation level induced by the input level equivalent to the activity level 30 min following EGF stimulation of Ras-GTP in E1 and E1/4 cells, respectively.

Application of the aforementioned selection process yielded two structures (numbers 4 and 29) that satisfied all criteria for model selection. In fact, the simulation results were quite similar for the two structures (see Figure 5A for the number 4 structure and Figure S4 for all structures). Interestingly, both structures possess positive feedback by ERK to B-Raf and negative cross-talk regulation by B-Raf to Raf-1, and the difference between the two structures being the absence or presence of ERK-mediated positive feedback regulation of Raf-1. However, we found that the ERK-mediated regulation of Raf-1 in number 29 structure was negligible since the maximum velocity was markedly smaller than that determined for Ras-GTP, indicating that the number 29 structure was essentially the same as the number 4 structure. Therefore, we concluded that the structure of the Raf-MEK-ERK cascade in E1 and E1/4 cells might be characterized by the number 4 structure, which possesses negative cross-talk regulation by B-Raf to Raf-1 and positive feedback by ERK to B-Raf (steps 14 and 16). The model description and kinetic parameters pertaining to the number 4 structure are provided in Tables S5 and S6.

### Characteristics of the estimated pathway structure of the Raf-MEK-ERK cascade

In order to investigate the characteristics of the identified pathway structure, we next carried out a steady-state analysis for ERK and B-Raf responses under the constant inputs of Ras- and Rap1-GTPs (Figures 5B and 5C). As a result, we found that ERK activity increased with a steep slope along with the input level of Ras-GTP coordination with ERK-B-Raf positive feedback that can stabilize the promotion of ERK activity, but be insensitive to that of Rap1-GTP (Figure 5B). This indicated that ERK activity might be regulated predominantly by Ras activity rather than Rap1 activity in this pathway structure. Interestingly, this conclusion was also true for B-Raf activity (Figure 5C), although B-Raf can also be regulated by Rap1-GTP. If this is the case, Rap1 activity would be less influential on ERK activity. In order to confirm the lesser impact of Rap1 activity on ERK activity, we compared ERK activity 5 min after 10 nM EGF treatment in the presence or absence of the PLCγ inhibitor U73122, which can indirectly suppress Rap1 activity in CHO cells (Tables 1, no. 7) [21]. U73122 showed less effect on ERK activity both in E1 and E1/4 cells, and is consistent with the above-mentioned perspective based on steady-state analysis.

As shown in Figure 4A, the peak level of Ras activation in E1/4 cells was considerably higher than that of E1 cells. We marked the points at which the amplitude of ERK and B-Raf activity would be induced by input levels equivalent to the peak values of Ras-GTP (Figures 5B and 5C) to determine the extent to which the differences impact on ERK and B-Raf activity. Since the input level of Rap1-GTP was not sensitive to ERK activity, we placed symbols at a zero input level of Rap1-GTP for clarity. Notably, the peak activity of Ras-GTP in E1/4 cells was placed at the upper level of ERK activity over the steep slope in comparison with that of E1 cells, which was placed at the lower level (Figure 5B). Therefore, we concluded that the experimentally observed difference in amplitude of ERK activity was mainly caused by differences in amplitude of Ras activity between E1 and E1/4 cells. Unlike the case of ERK, the peak activity of Ras-GTP in E1 cells was placed at the upper part of the slope of the B-Raf activity profile (Figure 5C); the amplitude of B-Raf activity is therefore quite close to that for E1/4 cells. Furthermore, we investigated ERK and B-Raf activities that would be induced by input levels equivalent to Ras activity 30 min following EGF treatment as depicted in Figure 4A. We found that B-Raf activity at 30 min differed markedly between E1 and E1/4 cells (Figure 5C), whereas ERK activity returned to considerably lower levels in both cell lines (Figure 5B). This implies that B-Raf activity would be sustained for a greater period of time in E1/4 cells than in E1 cells. Taken together, these results indicate that the characteristics of the Raf-MEK-ERK cascade are determined mainly by the amplitude of Ras activity and not Rap1 activity. Additionally, differences in the peak level of ERK activity between E1 and E1/4 cells might result from differences in Ras activation amplitude coordination with E1/4 cell-specific sustained B-Raf activation.

### Detailed upstream model of EGFR/ErbB4 heterodimer-mediated Ras activation

**Figure 6:**
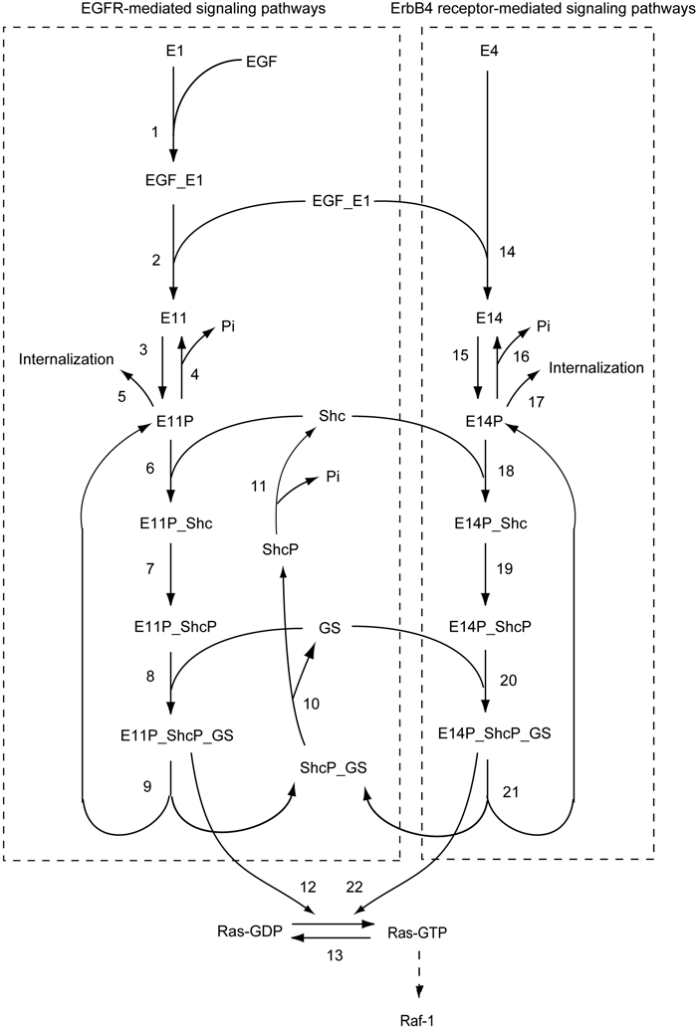
Detailed upstream model of EGFR/ErbB4 heterodimer-mediated Ras activation. The pathways in the left and right boxes are mediated by the EGFR homodimer and the EGFR/ErbB4 heterodimer, respectively. Ras activation is regulated by these two pathways via steps 12 and 22. When performing parameter estimation of total concentrations and kinetic parameters for the E1/4 cells, all parameters were estimated. On the other hand, the concentration of the ErbB4 receptors was set to zero for E1 cells, while other unknown parameters were considered to be identical as those for the E1/4 cells. Numbers shown correspond to the kinetic equations in Table S7.

**Figure 7:**
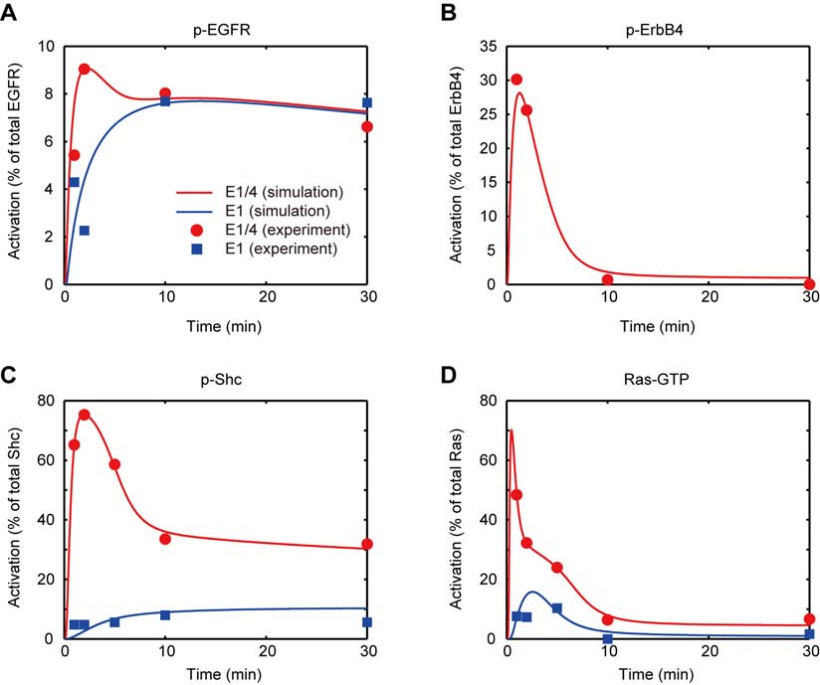
Time-course activity of signaling molecules with 10 nM EGF in ErbB signaling. The responses of the signaling molecules during 30 min are plotted with the corresponding experimental data listed in Table 1 (nos. 4 and 8–10). (A) Phosphorylated EGFR in E1 and E1/4 cells, respectively. (B) Phosphorylated ErbB4 receptor in E1/4 cells. (C) Phosphorylated Shc in E1 and E1/4 cells, respectively. (D) Ras-GTP in E1 and E1/4 cells, respectively. Blue and red lines indicate simulation data for E1 and E1/4 cells, respectively, and filled squares and circles indicate experimental data E1 and E1/4 cells, respectively. Graphs represent activity (% of total protein).

Many studies revealed that ErbB receptor-mediated Ras signaling is deeply involved in ERK activation upon EGF stimulation [31]. Interestingly, it was reported that Shc activity, which results in Ras activation [30], [32], might be regulated by mechanisms that differ between E1 and E1/4 cells [33]. However, the detailed mechanism concerning EGFR/ErbB4 heterodimer-mediated signaling is still unclear. In the previous section we demonstrated that differences in amplitude of ERK activity between the two cell lines might be caused predominantly by different Ras activities independently of Rap1 activation dynamics, where we assumed that the pathway structure and kinetic parameters of the central Raf-MEK-ERK cascade were the same. We therefore constructed a detailed upstream model of EGFR/ErbB4 heterodimer-mediated Ras activation to predict how differences in amplitude of Ras activity between E1 and E1/4 cells can be caused (Figure 6). We assumed that one of the crucial factors is the presence of the ErbB4 receptor in the pathways, implying that homodimer-mediated pathways (steps 1–12, the left box in Figure 6) are common in the two cell lines and that heterodimer-mediated pathways (steps 14–22, the right box in Figure 6) are E1/4 cell-specific. For a simulation of E1 cells, the concentration of the ErbB4 receptor was set to zero. Then, steps 14–22 were inactive and Ras activity could be controlled by only E11P_ShcP_GS (step 12), otherwise control was achieved through E11P_ShcP_GS and E14P_ShcP_GS (steps 12 and 22). Steps 1–11, which include Shc recruitment to the receptor, binding to the Grb2-SOS complex and receptor internalization, were adopted from the earlier study [26]. However, those were re-simplified to maintain the essential dynamics of the pathway [29]. Our experimental data indicated that the signal amplitude of EGFR phosphorylation was similar in E1 and E1/4 cells although the dynamics seemed to differ (Figure 7A), while the amplitude and dynamics of Shc phosphorylation were very different (Figure 7C). Therefore, we hypothetically introduced the steps 14–22 in order to take into account the effect of ErbB4 receptor-mediated signaling on Shc and Ras regulation. Kinetic parameters were also estimated using GLSDC (Genetic Local Search with distance independent Diversity Control) by comparing the experimental data (Table 1, nos. 4, 8–10) with simulated values (See Materials and Methods for parameter estimation of the upstream model). The model description and kinetic parameters are provided in Tables S7 and S8. When the simulation model was used to examine the effect of a 10 nM EGF perturbation on E1 and E1/4 cells, the model reasonably reproduced a similar signal amplitude of EGFR phosphorylation, different amplitude and dynamics of Shc phosphorylation, different amplitude of Ras activation, and transient dynamics of ErbB4 phosphorylation in E1/4 cells (Figure 7).

**Figure 8:**
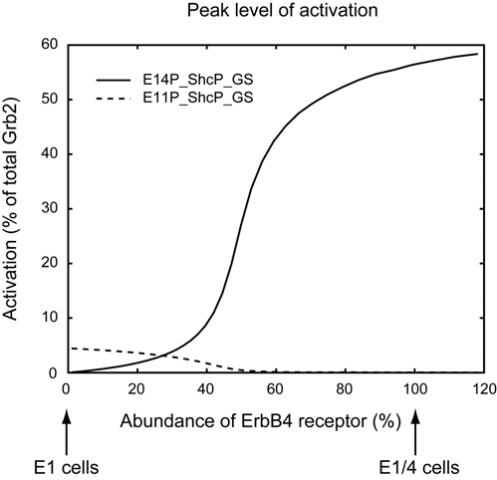
Effect of ErbB4 receptor concentration on E11P_ShcP_GS and E14P_ShcP_GS. The two complexes E11P_ShcP_GS and E14P_ShcP_GS in Figure 6 regulate Ras activity. The solid and dashed lines represent the change in peak activity (%) of E14P_ShcP_GS and E11P_ShcP_GS, respectively, following 10 nM EGF treatment and depending on the concentration of ErbB4 receptors in the detailed upstream model, respectively. The 0% ErbB4 receptor concentration (E4 = 0) is regarded as representing E1 cells, while the 100% level (E4 = 1.0) represents E1/4 cells.

**Figure 9:**
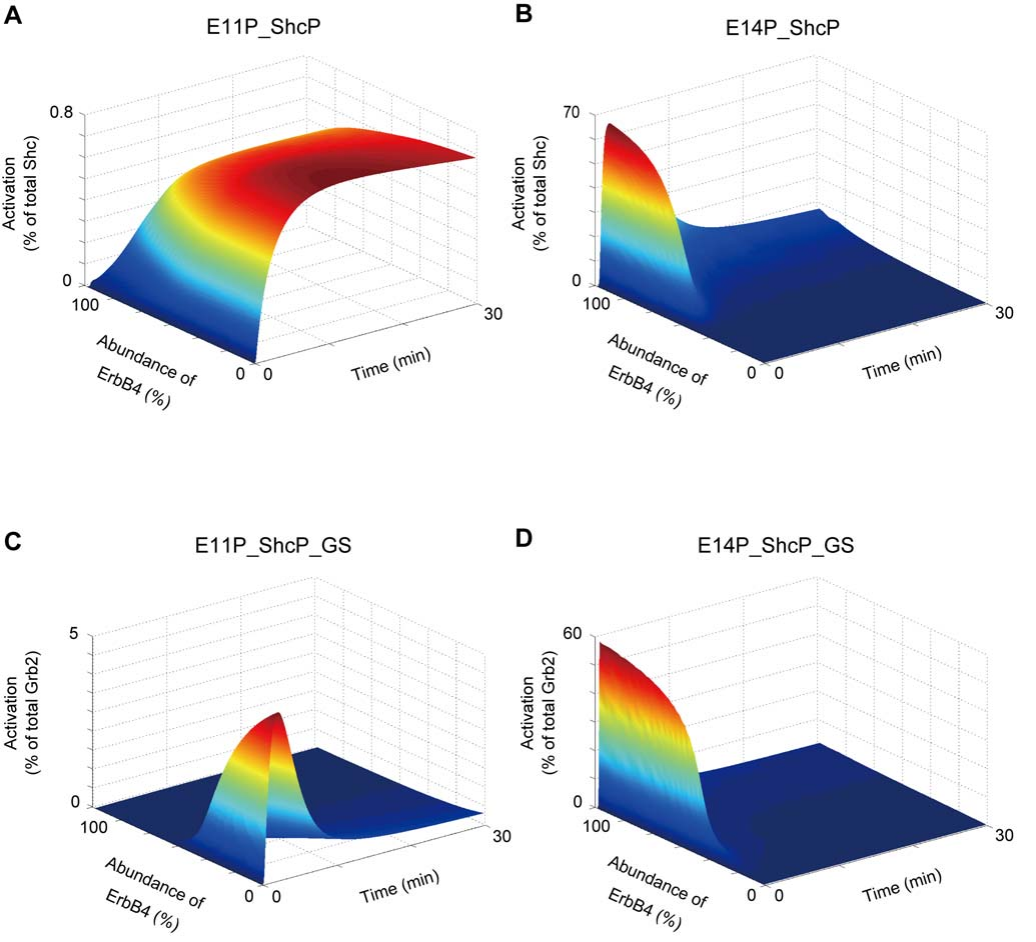
Effect of ErbB4 receptor concentration on the time-course pattern. The concentration of the ErbB4 receptor (E4) was changed from 100% of the total ErbB4 receptors (E4 = 1.0) in E1/4 cells to 0% (E4 = 0.0). Figures demonstrate time-course patterns of signaling molecules in relation to the abundance of the ErbB4 receptor in silico. (A) and (C) The signaling molecules E11P_ShcP and E11P_ShcP_GS in EGFR homodimer-mediated pathways, respectively. (B) and (D) The signaling molecules E14P_ShcP and E14P_ShcP_GS in EGFR/ErbB4 heterodimer-mediated pathways, respectively. The colors of the graphs indicate activity (% of total protein).

Next, we investigated the effect of ErbB4 receptor-mediated pathways on Ras activation. To this end, we varied the concentration of the ErbB4 receptor from about 100% of the total ErbB4 receptors in E1/4 cells to about 0% (Figures 8 and 9). The model with 0% ErbB4 receptors corresponds to the E1 cell model. As the abundance of ErbB4 receptors increased, the peak level of activation of E14P_ShcP_GS increased with a steep slope, whereas that of E11P_ShcP_GS decreased (Figure 8). Furthermore, we found that the peak level of E14P_ShcP_GS was much greater than that of E11P_ShcP_GS, especially for higher concentrations of the ErbB4 receptor. Therefore, the peak level of Ras activation might be critically affected by the concentration of ErbB4 receptors through E14P_ShcP_GS (step 22 in Figure 6) rather than E11P_ShcP_GS (step12). Figure 9 shows the time-course patterns of signaling molecules in this sensitivity analysis. The change in peak level of E14P_ShcP was also affected by the concentration of ErbB4 receptors as well as E14P_ShcP_GS (Figures 9B and 9D), although E11P_ShcP was not sensitive to such changes (Figures 9A). However, the peak level of E11P_ShcP_GS became quite small at around a 100% concentration of ErbB4 receptors despite such insensitivity (Figure 9C) compared to that of E14P_ShcP_GS (Figure 9D). E11P_ShcP and E14P_ShcP share the interaction partner, the GS (Grb2-SOS) complex. In addition, the initial reaction velocity of E14P_ShcP was faster than that of E11P_ShcP at higher concentrations of the ErbB4 receptor (60–100%) (Figures 9A and 9B). Therefore, GS might preferentially bind to E14P_ShcP rather than E11P_ShcP. These results as a whole indicate that the concentration of the ErbB4 receptor in our detailed model might be quite important for the considerably strong Ras activation observed in E1/4 cells, where the ErbB4 receptor-mediated signaling pathway can dominate Ras activation dynamics.

## Discussion

Cells co-expressing different ErbB receptors tend to undergo cellular transformation more frequently than cells expressing a single type of the receptor [3]–[6]. Our earlier study reported that cellular transformation occurs only in cells co-expressing both EGFR and ErbB4 receptors, but not in cells expressing only EGFR or ErbB4, suggesting that different cell fates might originate from the enhancement of ERK activation mediated by E1/4 cell-specific B-Raf activation, although homo- and hetero-dimers could recruit similar effector proteins upon EGF stimulation [21]. Therefore, a question is raised concerning how the signal amplitude of the signal transduction pathway and the cell-specific activation were controlled in the ErbB receptor co-expression system.

Since the Raf-MEK-ERK cascade is considered a core component of the signaling network, and Raf isoforms are key downstream targets of ErbB receptors [34], it is reasonable to assume that the Raf-MEK-ERK cascade consists of the same structure in E1 and E1/4 cells. However, since the regulation of Raf isoforms is complex and cell-specific [12], [18], [22], [35], we initially estimated the feedback/cross-talk structure of the cascade using topological modeling. In addition to the 6 measured quantities (Table 1, nos. 1–3), the time-course inputs of Ras- and Rap1-GTPs were also utilized as additional constraints in the determination (Table 1, nos. 4 and 5). The time-course patterns of Ras- and Rap1-GTPs (Figure 4) were sufficiently different to allow for a determination of parameters differentiating the Raf-1 and B-Raf pathways. Moreover, given the benefits of simplified modeling using minimal variation of feedback/cross-talk connections, we were able to arrive at a structure that possessed negative cross-talk regulation of Raf-1 by B-Raf and positive feedback by ERK to B-Raf. Since an earlier study indicated that ERK-induced phosphorylation of B-Raf on Thr^753^ promoted the disassembly of Raf-1/B-Raf heterodimer, followed by low kinase activity of Raf-1 in COS-1 cells [23], this result might support our estimated pathway structure. Activation and deactivation of Raf isoforms are tightly regulated, although the mechanisms involved in this regulation are not fully known. There are reports that B-Raf is activated by Ras and Raf-1 requires additional mediators [35], while 90% of ERK activity is dependent on B-Raf activity in nerve growth factor-induced PC12 cells [12]. On the other hand, contradictory studies showed that Raf-1 is activated by its own feedback from ERK [18] and that Raf-1 activates B-Raf in a Ras-dependent manner [22]. Our simulation analysis only explains topological regulation of time-course events, not the molecular functions. However, the ligand-stimulated Raf activation and deactivation mechanism seemed to involve cell- and ligand type-specific complex events. Therefore, our pathway estimation indicated that one can estimate the hidden regulatory pathways using mathematical modeling. In fact, it was reported that the feedback structure of the MAPK network can be either negative or positive depending on the kind of ligand, EGF or NGF, in PC-12 cells [36].

It is well known that ligand-induced activity of signaling proteins in a MAPK cascade can be promoted in an ultrasensitive manner depending on the pathway structure and kinetic parameters [24], [25]. From the steady-state analysis of ERK and B-Raf activity response to the constant input of Ras- and Rap1-GTPs, we found that ERK and B-Raf activity was steeply elevated along with the input level of Ras-GTP in the selected pathway structure, although there was no sensitivity to Rap-1. This lesser effect of Rap1 on ERK activity was also confirmed by Western blot analysis of ERK activation by suppressing the PLCγ-Rap1 pathway using the specific PLCγ inhibitor. The role of Rap1 in Ras-mediated signaling is still unclear. Rap1 might be implicated in both negative and positive control of Ras-mediated signaling depending on the kinds of ligand and cell lines [37], [38]–[40]. However, our results might be consistent with the earlier results of Zwartkrius et al. [41] in which it was shown that Rap1 did not affect ERK activation in Rat-1 cells.

Our earlier study showed that E1/4 cells induced specific B-Raf and higher ERK activation upon EGF stimulation than E1 cells, followed by cellular transformation [21]. From the steady-state analysis, we concluded that the peak level of Ras activity in E1/4 cells was sufficiently high to induce the ultrasensitive ERK activation. On the other hand, activity in E1 cells is near the bottom of the slope of the Ras-ERK steady-state response curve, such that the amplitude of ERK activity in E1 cells became considerably low. In addition, the B-Raf steady-state response induced by the peak level of Ras activity is similar and high in E1 and E1/4 cells, but considerably different when evaluating Ras activity 30 min after EGF stimulation. Such a difference might cause the different B-Raf activation dynamics upon EGF stimulation, which are sustained in E1/4 cells and transient in E1 cells. The sustained B-Raf activation can then stabilize the promotion of ERK activity through the ERK-B-Raf positive feedback loop in E1/4 cells.

The mechanism of Ras activation dynamics has been investigated in many studies using mathematical modeling [26], [29], [42]. However, less is known about the effect of heterodimerization on signal transduction pathways. In the present study, we found that the amplitude of Ras activity becomes more potent as the concentration of ErbB4 receptors increases in E1/4 cells. An earlier study revealed that co-expression of EGFR with ErbB2 or ErbB3 biases signaling to the cell surface and retards signal down-regulation, followed by prolonged signaling of downstream molecules [43]. Unlike the ErbB1-3 system, we speculated that the initial reaction velocity of EGFR/ErbB4 receptor heterodimerization might be important and considerably higher than that of EGFR homodimerization in our CHO cells. In fact, the critical parameter in determining signal efficacy for the EGFR homodimer-mediated signal transduction system is the initial velocity of receptor activation [42]. We have reached a similar conclusion with EGFR/ErbB4 heterodimer-mediated signaling, but here we insisted that the initial velocity of heterodimerization might be more effective for Ras activation dynamics than that of the homodimer. If we follow the ODEs of the detailed upstream model, the concentration of dimers, E11 and E14, are modulated by the following ODEs:
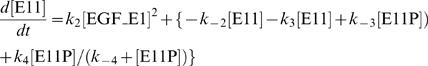
(8)

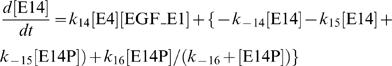
(9)Since the initial concentrations of E11, E11P, E14 and E14P are equivalent to zero, the initial velocities of E11 and E14 are dominated by the first terms. Therefore, in the range
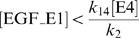
(10)the reaction velocity of E14 becomes higher than that of E11, followed by the difference in the initial reaction velocity. Again, it should be noted that such a range would be wider when the concentration of ErbB4 receptors is high.

## Materials and Methods

### Materials

Recombinant human epidermal growth factor (EGF) was purchased from PeproTech House (London, England). Antibodies for detecting phospho-p44/42 ERK, ERK phospho-MEK and MEK were purchased from Cell Signaling Technology, Inc. (Beverly, MA). Anti-EGFR receptor, anti-ErbB4 receptor, anti-Rap1, anti-Raf and anti-phosphotyrosine (PY20) antibodies were obtained from Santa Cruz Biotechnology (Santa Cruz, CA). Anti-Ras antibody was purchased from BD Biosciences (San Jose, USA). GST-Ral GDS-Rap binding domain (RDB) agarose, Raf-Ras binding domain (RDB) agarose, anti-Shc, and anti-phospho-Shc (Tyr317) antibodies were purchased from Upstate Biotechnology (Lake Placid, NY). U73122 (PLCγ inhibitor) and U0126 (MEK inhibitor) were obtained from Calbiochem (San Diego, CA). Methods for constructing Chinese hamster ovary (CHO) cells expressing full-length human EGFR and both EGFR and ErbB4 receptors are described elsewhere [21], [44].

### Cell Culture

CHO cells expressing EGFR or EGFR and ErbB4 receptors were routinely maintained in DMEM/F12 (Gibco BRL, Githersburg, MD) medium supplemented with 10% bovine calf serum and antibiotics. For detection of the effect of growth hormones, the cells were starved in serum-free DMEM/F12 for 16–24 hours prior to the experiment. To test the effect of kinase and phosphatase inhibitors, the cells were pretreated with the inhibitors 10 min prior to the addition of EGF. The concentration of EGF for cell treatments is 10 nM unless otherwise indicated.

### Western Blot Analysis

The protein-protein interactions and protein phosphorylation levels were measured by Western blot analysis. For protein-protein interaction or receptor phosphorylation analysis, proteins were immunoprecipitated with corresponding antibodies and immunoblotted with antibodies for their interacting proteins or anti-phosphotyrosine antibody. We examined ERK phosphorylation as a downstream marker of the MAPK cascade. We used Raf-RDB and a Rap1 pull-down assay as activation markers for Ras and Rap1, respectively, as described earlier [21]. The band intensities of proteins were quantified using a densitometer (Fuji Film Corp, Tokyo, Japan) and normalized by dividing the signal intensity at each time point by the control at 0 min, and then subtracting 1.0 from these values (Table S1), thus generating normalized data with no units. For the B-Raf kinase assay, E1/4 cells were treated with 10 nM EGF for 1, 2, 5, 10 and 30 min. Cell lysate was immunoprecipitated (IP) with an anti-B-Raf antibody and incubated with a MEK substrate. MEK phosphorylation was examined with an antibody against phospho-(Ser218/222) MEK1 and phospho-(Ser222/226) MEK2 followed by densitometric quantification.

### Construction of the Raf-MEK-ERK cascade model

The model was constructed on the basis of observations made in earlier studies. Raf-1 activity is promoted by the association with an activated form of Ras-GTP (step 1 in Figure 2) and reduced by phosphorylation on Ser^259^ (step 2) [45], [46]. B-Raf can be activated through PLCγ and subsequent small GTPase Rap1 (step 3) [37], [41]. MEK is directly activated by Raf-1 and B-Raf (steps 5 and 6) [31], and then doubly phosphorylates ERK (steps 8 and 9) [25], [28]. The MAPK cascade can be negatively modulated by protein phosphatase 2A (PP2A) through dephosphorylation of MEK (step 7) and by MAPK phosphatase 3 (MKP3) through dephosphorylation of ERK (steps 10 and 11) [47], [48]. Steps 12–19 were prepared as possible connections regarding feedback/cross-talk regulations.

### Procedure of model selection based on parameter estimation

For each of the 29 candidate structures, we initially performed three rounds of parameter estimation whilst changing the starting points for the parameter search. As an estimator, we used the genetic algorithm GLSDC [49]. The error equation that is optimized by GLSDC, *ERR*, was defined by the total sum of squares relating the gap between the experimental data and simulated values at each time point as follows:
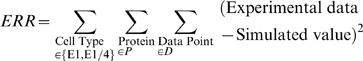
(1)where the sets *P* = {p-ERK, p-MEK} and *D* are obtained from Table S1 (nos. 1 and 2). Additionally, we imposed a quantitative effect relating to U0126 for each candidate as a constraint on the estimator. U0126 is an irreversible inhibitor of MEK and functionally lowers the maximum velocity of this enzyme. We set the velocity (steps 8 and 9 in Figure 2) to 0 to represent complete inhibition. Then the peak level of simulated MEK activation with *V*_8_ = *V*_9_ = 0 should be lower than that of the experimental data with U0126 (Table S1, no. 3). The following constraints were therefore imposed during estimation for E1 and E1/4 cells, respectively:
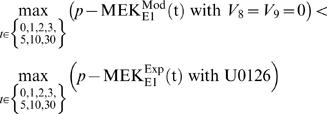
(2)

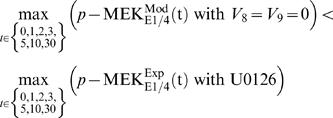
(3)With this setting, the total concentration of each protein equals unity. We assumed that all proteins are inactive prior to EGF simulation. We then evaluated each of the estimated parameters using the quantitative criteria (1)–(3) as outlined below, and selected one that satisfied most of the criteria of the three trials. If the number of satisfied criteria was identical, one that yielded the smallest estimation error was selected. The criteria for model selection were defined as follows:

1. Estimation error

For each data point ∈ *D*

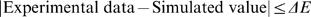
(4)where
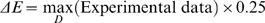
(5)Set *D* is obtained from Table S1 (nos. 1 and 2). The 25% of the maximum experimental value was used as a threshold since the model describes topological regulation rather than detailed molecular mechanism, and might not result in perfect fitting.

1. Effect of U0126 (the inequalities (2) and (3))
2. Sustained B-Raf activation in E1/4 cells
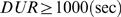
(6)where *DUR* represents the duration time calculated from the time-course data generated using the model for E1/4 cells, and defined by
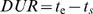
(7)
*t_s_* represents that time point at which B-Raf activity exceeds 70% of the maximum activity, and *t_e_* represents that time point after *t_s_* at which B-Raf activity becomes lower than 70% of the maximum activity. If *DUR* values are present due to oscillatory behavior, the maximum one is used. From the experimental results, the threshold was set to 1000 sec (Table S1, no. 6).

### Parameter estimation of upstream model

The error equation in GLSDC was defined by Eq. (1) with *P* = {p-EGFR, p-Shc, Ras-GTP, p-ErbB4} and *D* as shown in Table S1 (nos. 4, 8–10). The search range for an estimated parameter was limited within a neighbor of the corresponding value provided in the earlier study [26] (Table S9). Additionally, we assumed that the parameter values associated with step nos. 1–12 were identical for E1 and E1/4 cells. Since ShcP-GS bound to EGFR regulates Ras activation in both step nos. 12 and 22, the parameter value of step no. 22 was considered to be identical to that of step no. 12 for simplicity, although the kinetic parameter might not be necessarily the same given the different dimer partners (EGFR or ErbB4). Use of these constraints facilitated selection during the course of the fitting. Under these conditions, we performed ten rounds of parameter estimation to reproduce the experimental data (Table 1, nos. 4, 8–10) since the upstream model seemed to be more complex than the topological Raf-MEK-ERK model. Finally, the parameter that yielded the smallest estimation error was selected.

### Model Development

To describe the biochemical reactions and connectivity of signaling molecules in this study, we adopted a deterministic ordinary differential equation (ODE) model. This methodology has been employed in many studies using the law of mass action and the Michaelis-Menten equation [24], [26], [29], [42]. The model was created to reproduce the normalized experimental data mentioned in Materials and Methods and was implemented with MATLAB 5.1 (The Mathworks, Inc.) on an AMD Opteron 2.2GHz workstation running SuSE Linux Enterprise Server 9 (Novell). The MATLAB function “ode15s” was applied to solve the ODE (http://www.mathworks.com/ access/helpdesk/help/techdoc/ref/ode15s.html). All code is available upon request. As a model parameter estimator, we used the genetic algorithm GLSDC [49]. The program was run on the RIKEN Super Combined Cluster (RSCC) system.

## Supporting Information

Text S1 (0.03 MB DOC)

Table S1 (0.03 MB XLS)

Table S2 (0.02 MB XLS)

Table S3 (0.02 MB XLS)

Table S4 (0.03 MB XLS)

Table S5 (0.02 MB XLS)

Table S6 (0.02 MB XLS)

Table S7 (0.02 MB XLS)

Table S8 (0.02 MB XLS)

Table S9 (0.03 MB XLS)

Figure S1 The effect of MEK inhibitor U0126 on ERK phosphorylation in E1 and E1/4 cells. Serum-starved E1 and E1/4 cells were incubated with 10 nM EGF for the indicated time period with or without pretreatment of 200 nM U0126. ERK phosphorylation was analyzed by Western blot with the corresponding anti-phospho-specific ERK antibodies (upper panel), and then reblotted with an anti-ERK antibody (lower panel). (A) Western blot for E1 cells. (B) Western blot for E1/4 cells. Data show a representative figure of three independent experiments.
(1.67 MB TIF)

Figure S2 Transfer function model of the signaling pathways. (A) The substrate S is activated by E_act_ and deactivated by E_deact_. The activator E_act_ and deactivator E_deact_ can be indirectly activated by ligand. (B) Figure shows a practical example of (A). When the substrate S is Raf-1, the activator E_act_ corresponds to Ras-GTP that is indirectly activated by EGF through some signaling molecules such as Shc, Grb2, and SOS. (C) The intermediate reactions between L and E_act_ (E_deact_) are approximated by the first-order transfer function with the time constant T_1_ (T_2_) and the system gain G_1_ (G_2_).
(0.72 MB TIF)

Figure S3 Model of input signal generator for Ras- and Rap1-GTPs. The input signal generator reproduces the time-course data of Ras- and Rap1-GTPs with 10 nM EGF. The model is constructed with eight transfer functions (steps 20–27). The outputs of the transfer functions regulate the activity of S_1_, S_2_, S_3_ and S_4_, which are activators or deactivators for Ras and Rap1 (steps 28–35). Ras and Rap1 activity is then regulated by those components (steps 36–39). The symbols are summarized in Table S2. Numbers shown correspond to the kinetic equations in Table S3.
(0.61 MB TIF)

Figure S4 Fitting results of the 29 structures. This Figure contains 29×4 figures where row corresponds to a structure number, and column activated proteins. Blue and red lines (markers) indicate simulation (experimental) results of E1 and E1/4 cells, respectively. If a structure satisfied criteria (1)–(3) of the main text, the word “pass” was put on the upper side of each figure, otherwise “fail” was put on there. Error bar indicates the upper and lower bounds calculated from criterion (1) of the main text. A value on the upper side of a figure in column 4 shows the duration time. Green and magenta colors mean “pass” and “fail”, respectively. The *x*- and *y*-axes represent time (min) and activation (% of total protein), respectively.
(0.34 MB PDF)
