# Supplementary material for: Topological Analysis of MAPK Cascade for Kinetic ErbB Signaling
Source: PLoS One. 2008 Mar 12;3(3):e1782. doi: 10.1371/journal.pone.0001782 (PMC2262155; doi:10.1371/journal.pone.0001782)
Supplement: Text S1 — (0.03 MB DOC) [file pone.0001782.s001.doc]

Text S1.

Basic idea of transfer function model

Figure S2A shows an enzymatic reaction for a substrate S elicited by ligand stimulation, in which activator E_act_ and deactivator E_deact_ are indirectly stimulated by ligand L. For example, in the case of S being the Raf-1 protein in MAPK signal transduction, the activator E_act_ corresponds to Ras-GTP (Figure S2B). The activation of Ras is not directly catalyzed by EGF, but contains several biochemical processes including receptor dimerization, recruitment of adaptor proteins, etc. In order to describe the precise reactions for Ras activation, we have to direct considerable effort towards the modeling. However, it is convenient to approximate intermediate reactions using linear dynamics if observed reactions do not contain high-order dynamics (Figure S2C). The approximation by first-order differential equation provides the two essential elements of dynamics, time constant T and system gain G. The first-order differential equation can be converted into a transfer function, and the two fundamental parameters explicitly appear in the form of a transfer function. In keeping with this approach, the dynamics in Figure S2C is described by the following equations:

$$\begin{matrix} \frac{d[\text{S}]}{dt}=-\frac{V_{1}[\text{E}_{\text{act}}][\text{S}]}{K_{m1}+[\text{S}]}+\frac{V_{2}[\text{E}_{\text{deact}}][\text{S*}]}{K_{m2}+[\text{S*}]} \\ \frac{d[\text{S*}]}{dt}=\frac{V_{1}[\text{E}_{\text{act}}][\text{S}]}{K_{m1}+[\text{S}]}-\frac{V_{2}[\text{E}_{\text{deact}}][\text{S*}]}{K_{m2}+[\text{S*}]} \end{matrix}$$

where [E_act_] ([E_deact_]) is the concentration of activator (deactivator), T_1_ (T_2_) denotes time constant that represents the speed of activation, and G_1_ (G_2_) denotes system gain that represents the magnitude. [L] is the concentration of ligand.

Application to input signal generator

In order to simulate the model, we need to input the time courses of 10 nM EGF-stimulated Ras-GTP and Rap1-GTP into the ODE models. Here, we developed the input signal generator to mimic the dynamics of Ras and Rap1 activation in E1 and E1/4 cells. For simplicity, the input generator was developed with a first order transfer function model (TFM) rather than an interpolation function of the experimental data of Ras- and Rap1-GTPs (Figure S3). The biological meaning of TFM is that EGF stimulation is transmitted to Ras regulation (steps 36 and 37) through a Ras activator (S_1_*; steps 20, 21, 28 and 29) and deactivator (S_2_*; steps 22, 23, 30 and 31). Likewise, Rap1 (steps 38 and 39) is regulated by a Rap1 activator (S_3_*; steps 24, 25, 32 and 33) and deactivator (S_4_*; steps 26, 27, 34 and 35). The TFM parameters were determined with GLSDC by comparing experimental data of Ras- and Rap1-GTPs with simulated values (Table 1, nos. 4 and 5). The model description is provided in Table S2-S4.
