# Supplementary figures and images for: Topological Analysis of MAPK Cascade for Kinetic ErbB Signaling
Source: PLoS One. 2008 Mar 12;3(3):e1782. doi: 10.1371/journal.pone.0001782 (PMC2262155; doi:10.1371/journal.pone.0001782)

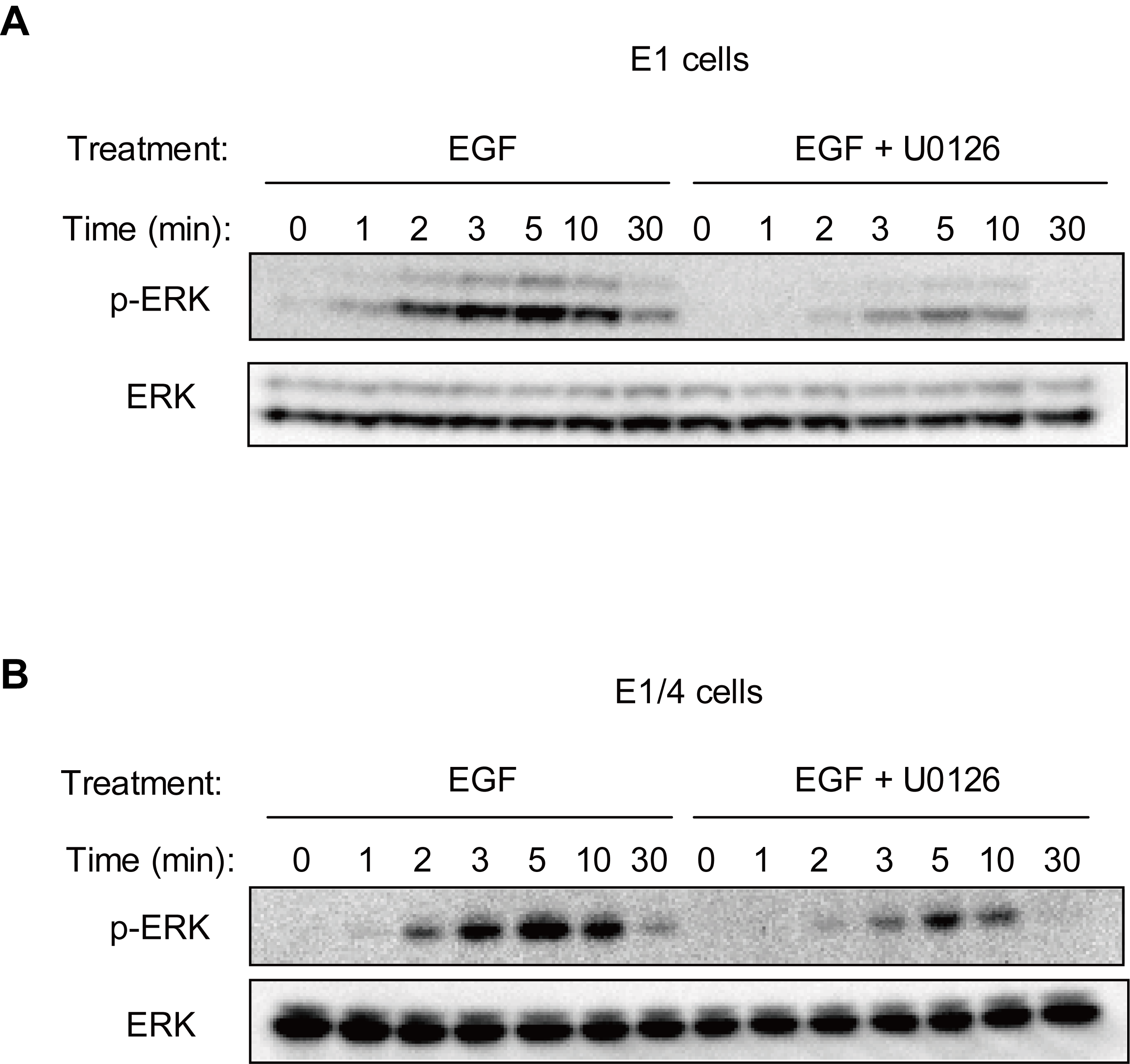

Supplement: Figure S1 — The effect of MEK inhibitor U0126 on ERK phosphorylation in E1 and E1/4 cells. Serum-starved E1 and E1/4 cells were incubated with 10 nM EGF for the indicated time period with or without pretreatment of 200 nM U0126. ERK phosphorylation was analyzed by Western blot with the corresponding anti-phospho-specific ERK antibodies (upper panel), and then reblotted with an anti-ERK antibody (lower panel). (A) Western blot for E1 cells. (B) Western blot for E1/4 cells. Data show a representative figure of three independent experiments. (1.67 MB TIF) [file pone.0001782.s011.tif]

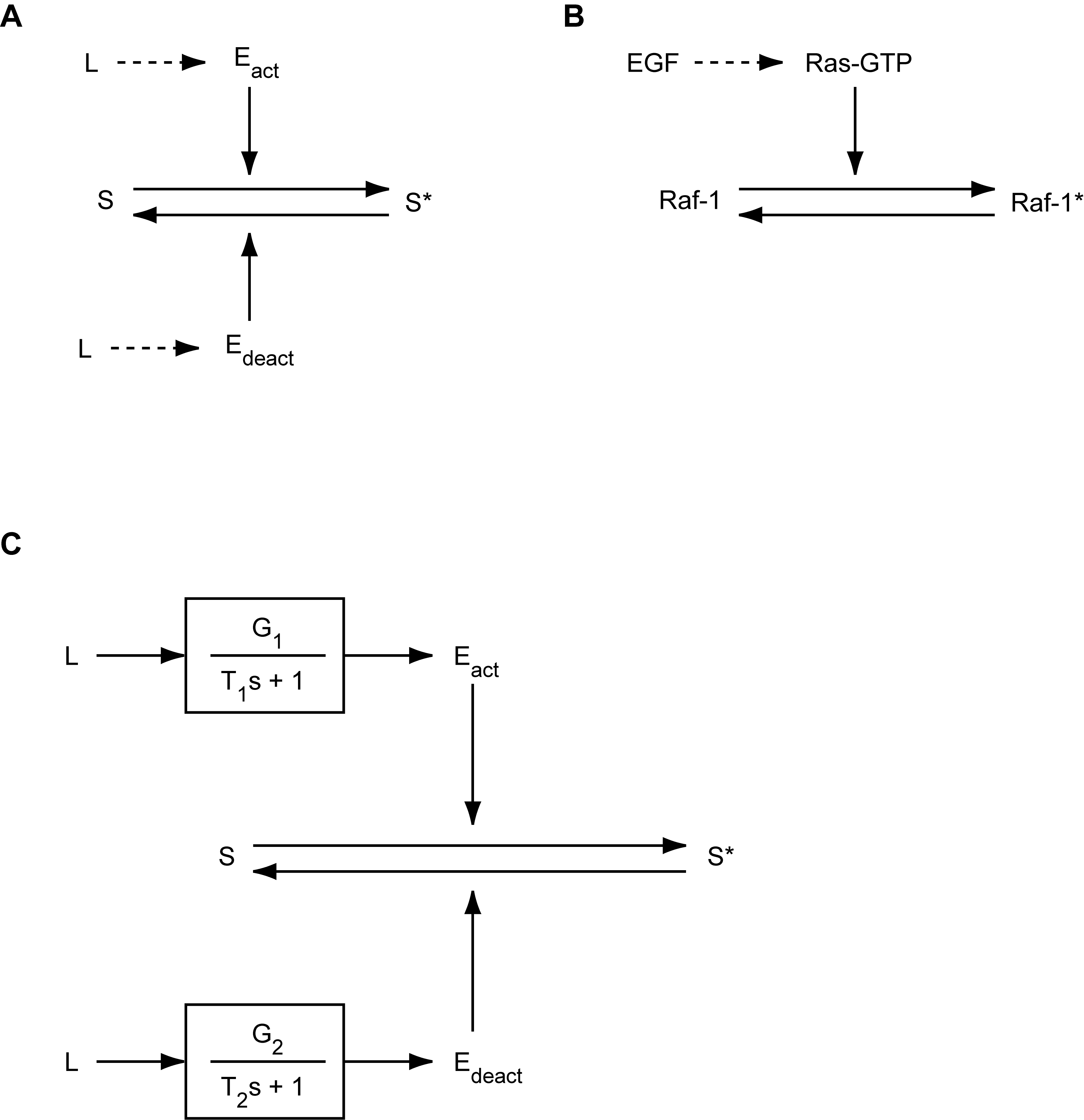

Supplement: Figure S2 — Transfer function model of the signaling pathways. (A) The substrate S is activated by Eact and deactivated by Edeact. The activator Eact and deactivator Edeact can be indirectly activated by ligand. (B) Figure shows a practical example of (A). When the substrate S is Raf-1, the activator Eact corresponds to Ras-GTP that is indirectly activated by EGF through some signaling molecules such as Shc, Grb2, and SOS. (C) The intermediate reactions between L and Eact (Edeact) are approximated by the first-order transfer function with the time constant T1 (T2) and the system gain G1 (G2). (0.72 MB TIF) [file pone.0001782.s012.tif]

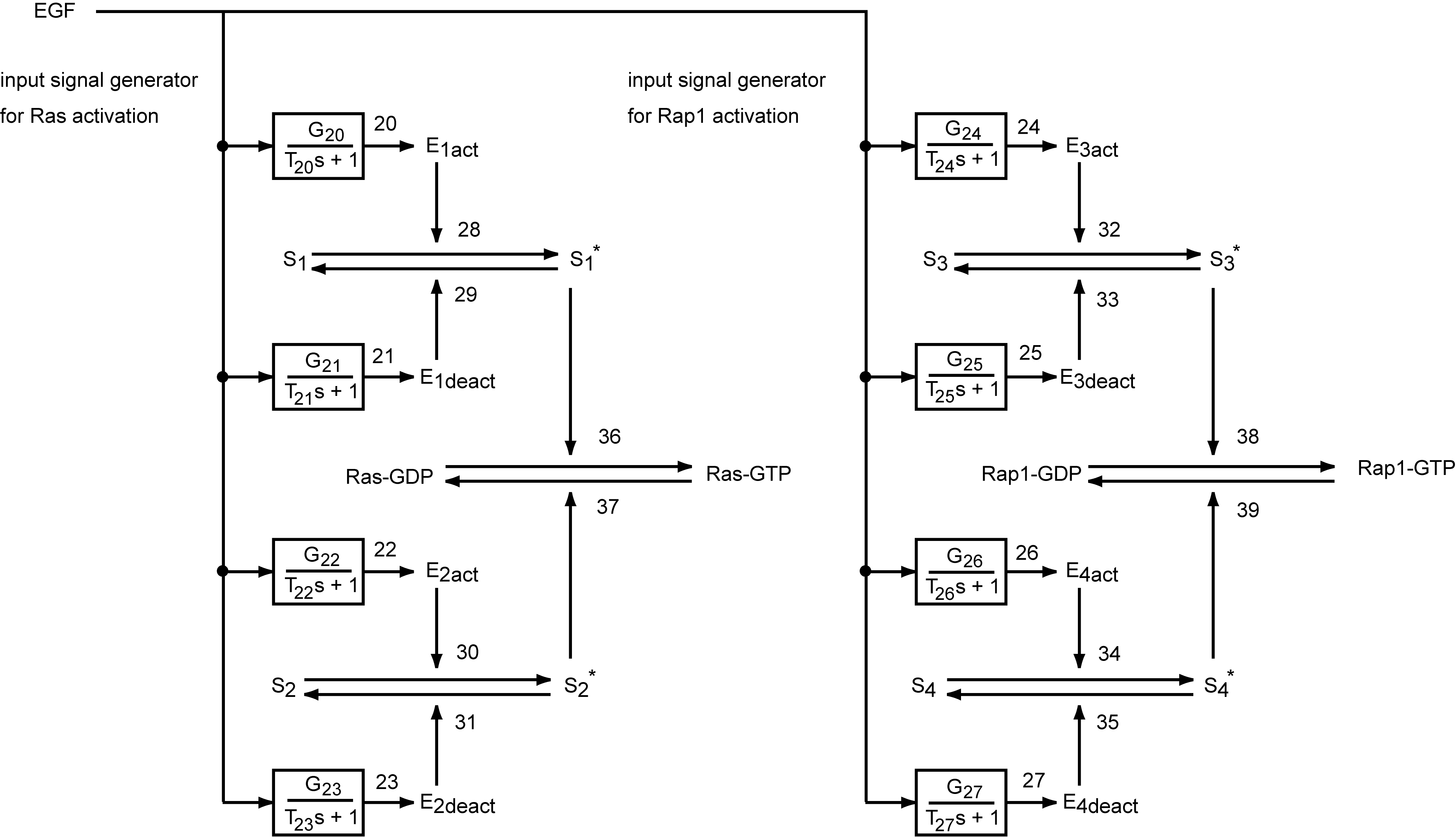

Supplement: Figure S3 — Model of input signal generator for Ras- and Rap1-GTPs. The input signal generator reproduces the time-course data of Ras- and Rap1-GTPs with 10 nM EGF. The model is constructed with eight transfer functions (steps 20–27). The outputs of the transfer functions regulate the activity of S1, S2, S3 and S4, which are activators or deactivators for Ras and Rap1 (steps 28–35). Ras and Rap1 activity is then regulated by those components (steps 36–39). The symbols are summarized in Table S2. Numbers shown correspond to the kinetic equations in Table S3. (0.61 MB TIF) [file pone.0001782.s013.tif]

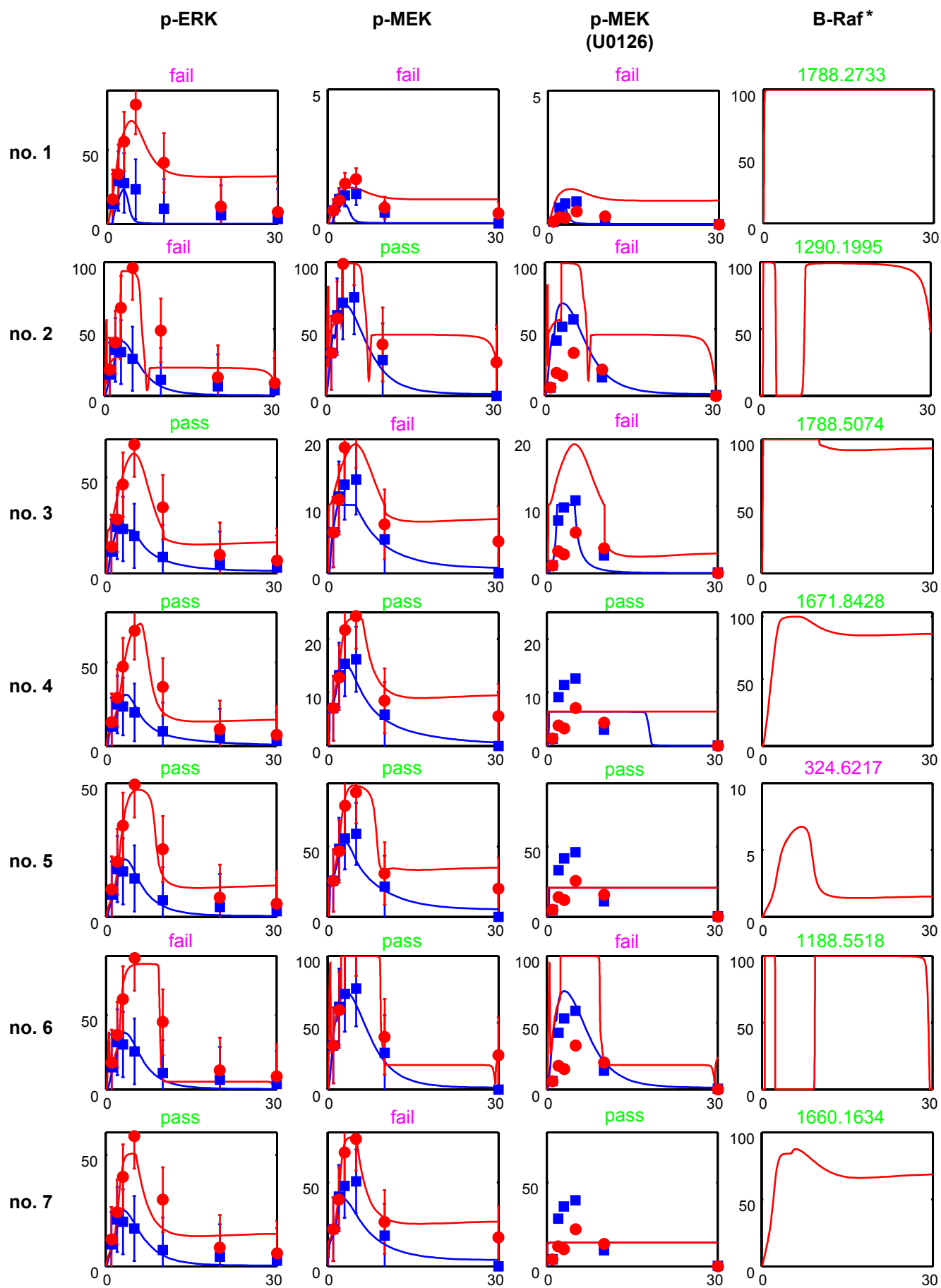

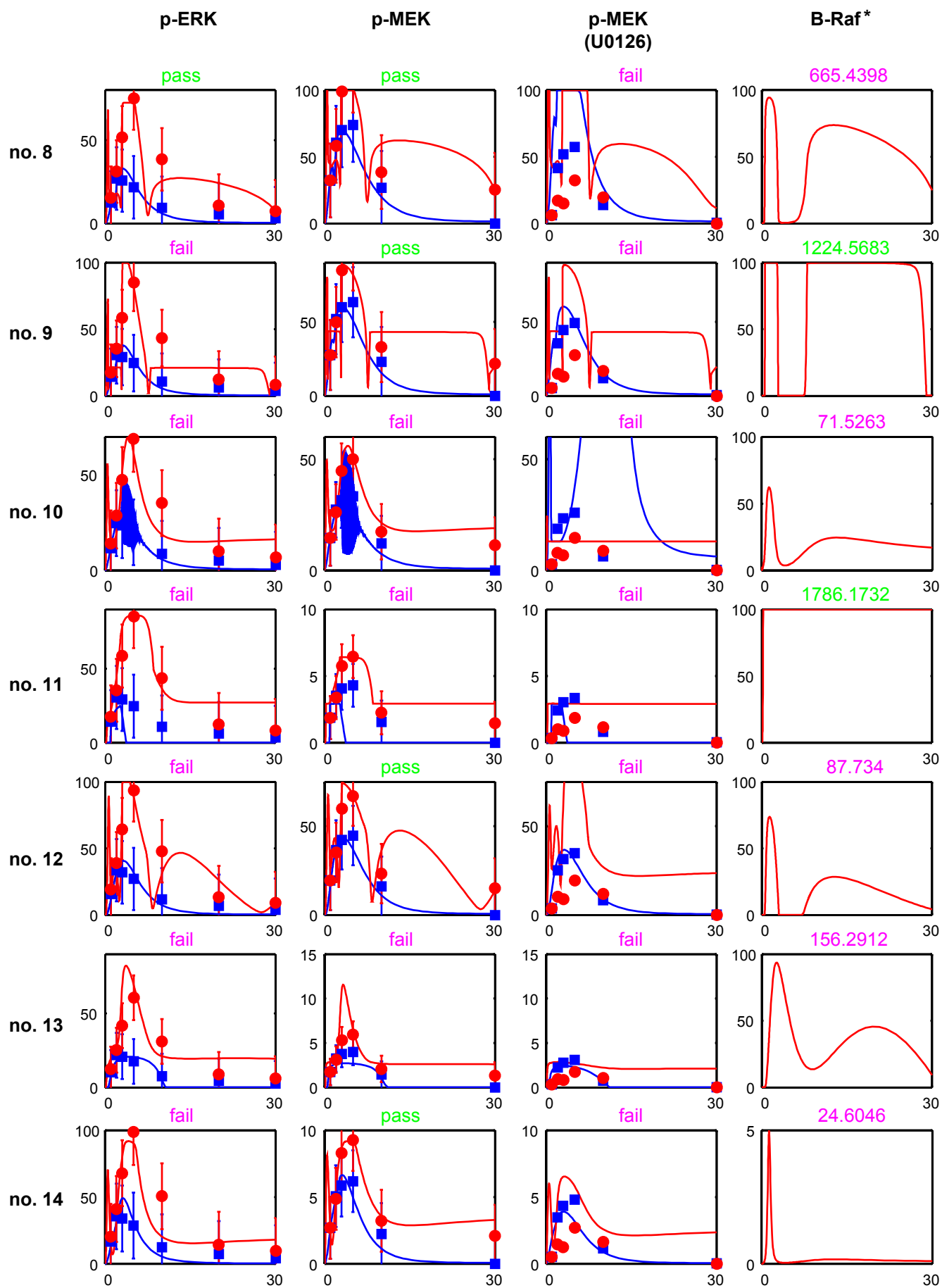

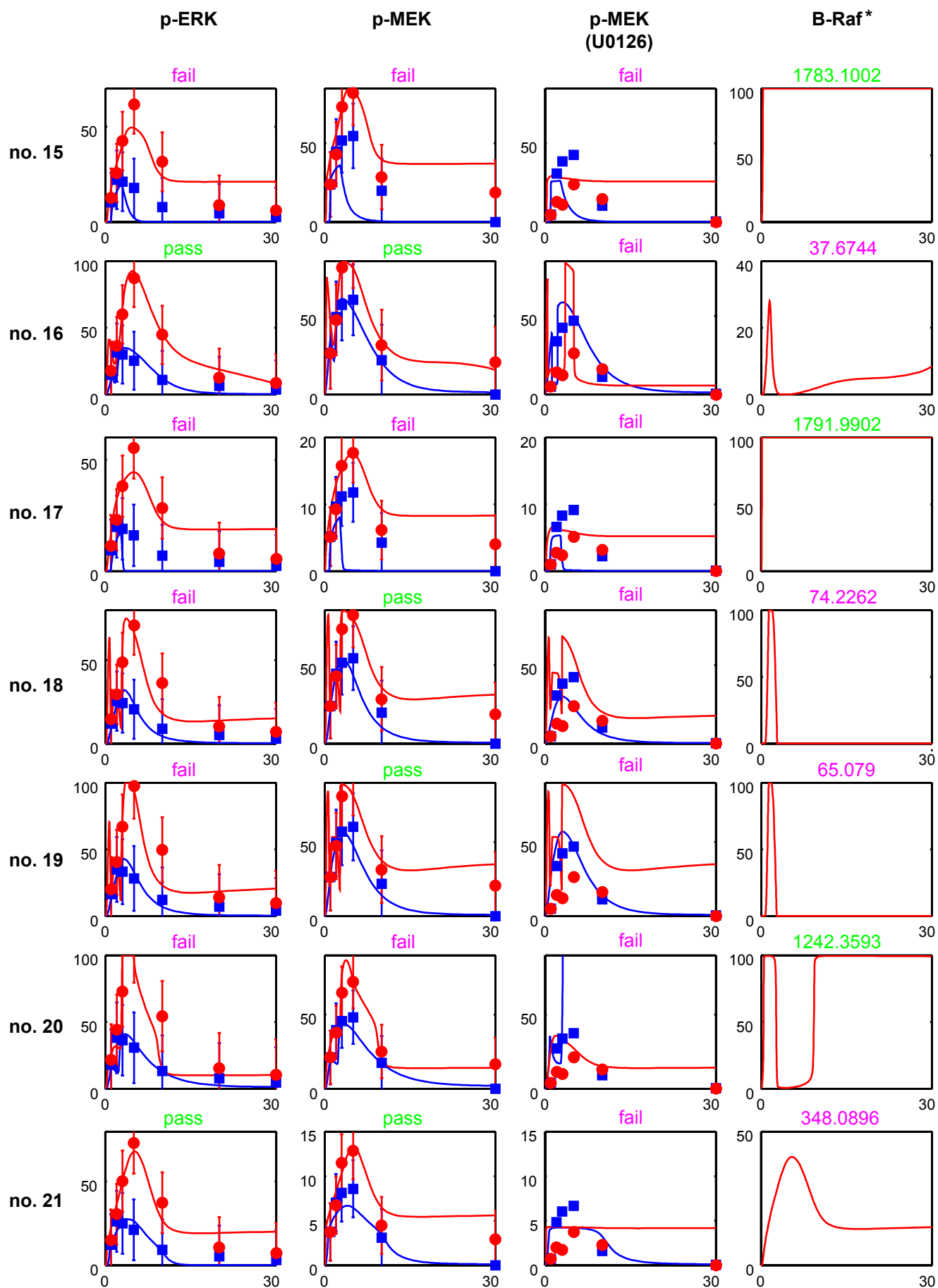

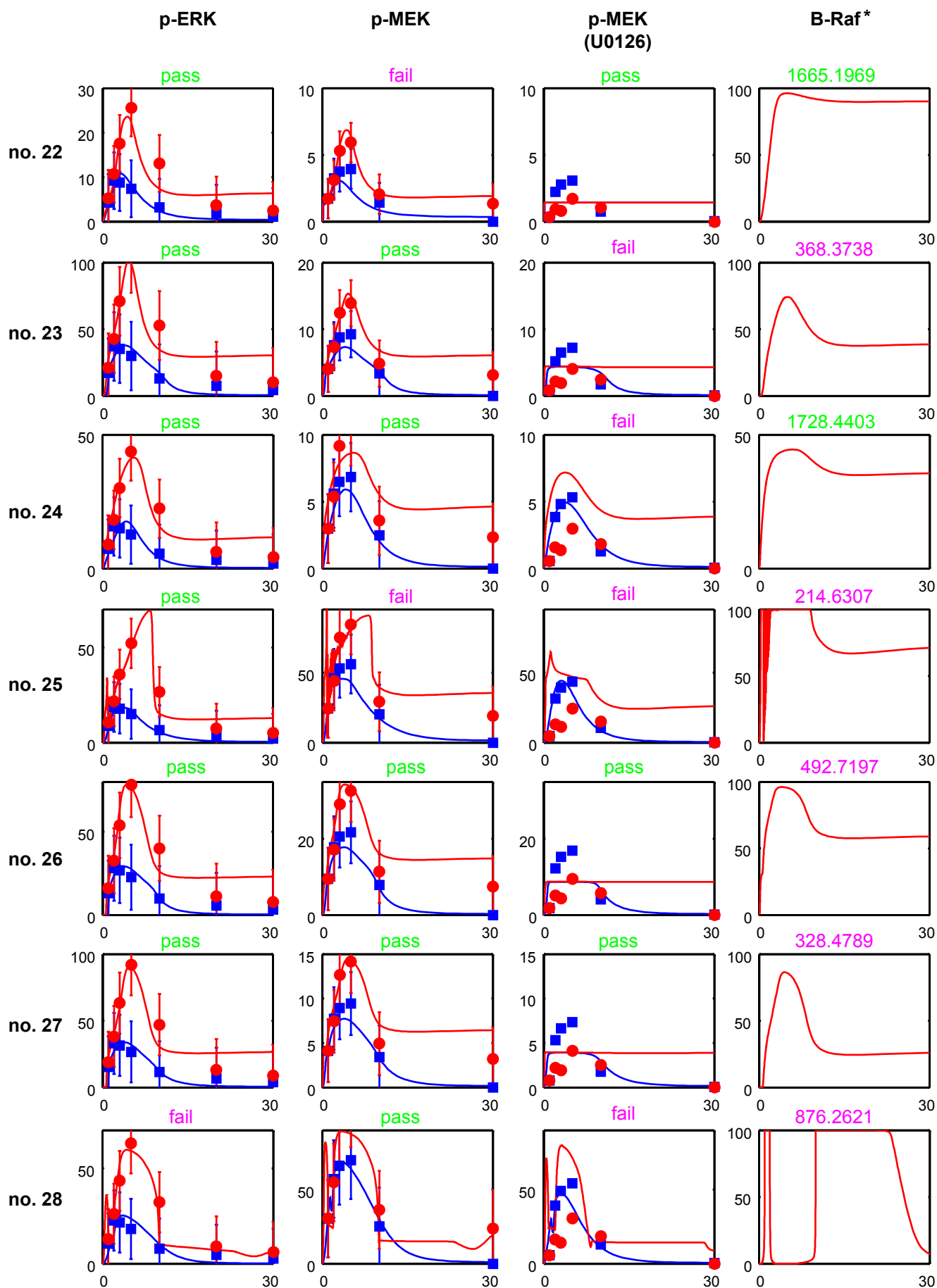

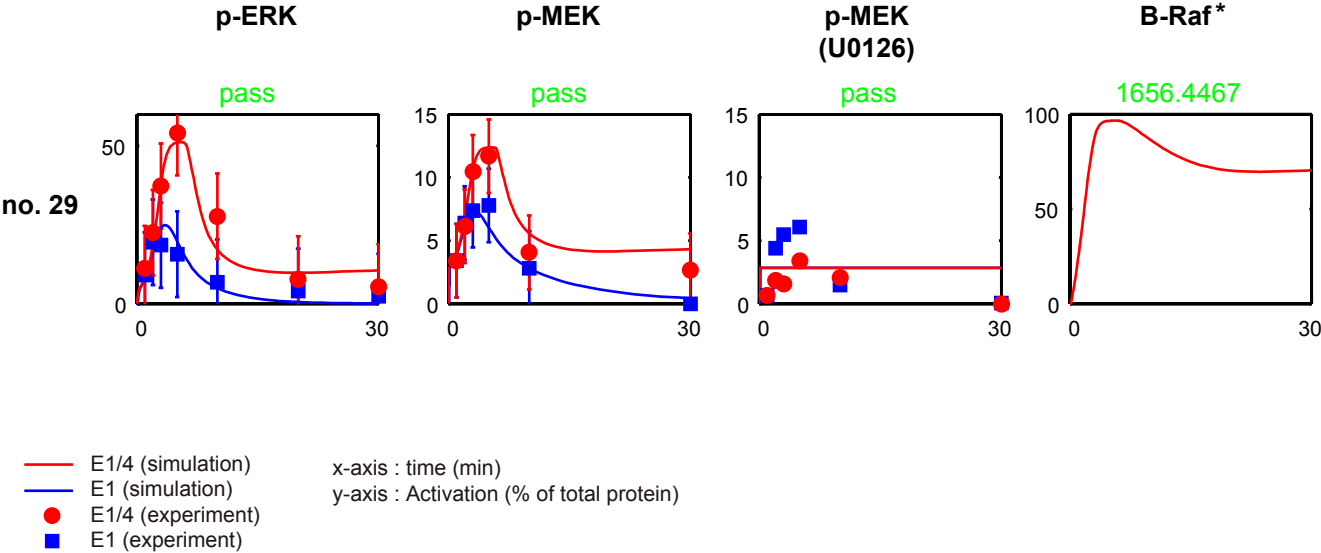

Supplement: Figure S4 — Fitting results of the 29 structures. This Figure contains 29×4 figures where row corresponds to a structure number, and column activated proteins. Blue and red lines (markers) indicate simulation (experimental) results of E1 and E1/4 cells, respectively. If a structure satisfied criteria (1)–(3) of the main text, the word “pass” was put on the upper side of each figure, otherwise “fail” was put on there. Error bar indicates the upper and lower bounds calculated from criterion (1) of the main text. A value on the upper side of a figure in column 4 shows the duration time. Green and magenta colors mean “pass” and “fail”, respectively. The x- and y-axes represent time (min) and activation (% of total protein), respectively. (0.34 MB PDF) [file pone.0001782.s014.pdf]
